# Multiplex ligation-dependent probe amplification workflow for the detection of submicroscopic chromosomal abnormalities in patients with developmental delay/intellectual disability

**DOI:** 10.1186/1755-8166-6-7

**Published:** 2013-02-06

**Authors:** Leona Morozin Pohovski, Katja K Dumic, Ljubica Odak, Ingeborg Barisic

**Affiliations:** 1 Department of Pediatrics, Division of Medical Genetics, Children's Hospital Zagreb, Medical School University of Zagreb, Klaiceva 16, 10000, Zagreb, Croatia

**Keywords:** Intellectual disability, Chromosome aberrations, Genetic testing, Developing countries

## Abstract

**Background:**

Array based comparative genomic hybridization (arrayCGH) has been increasingly used as the method of choice for diagnosis of patients with unexplained developmental delay/intellectual disability (DD/ID) but is not universally available for the high throughput use in routine practice. The next-generation sequencing (NGS) techniques, emerging as a new tool in clinical diagnostics, are at present quite labour-intensive and expensive. Since multiplex ligation-dependent probe amplification (MLPA) is relatively fast, easily interpreted and cost-effective, it is still a method of choice for screening large cohorts of patients with DD/ID.

**Results:**

We prospectively studied a cohort of 150 patients with DD/ID with or without dysmorphic features or additional congenital abnormalities. We used two distinct MLPA kits, SALSA P036 and P070, for subtelomere screening and MLPA kit SALSA P245 for the 21 common microdeletion syndromes. Subtelomere analysis was performed by both kits in all patients. All imbalances were verified by follow-up MLPA kits. The MLPA analysis revealed chromosome aberrations in 21 (14%) cases: 11 subtelomeric rearrangements and 10 microdeletions.

**Conclusions:**

We have presented the results of the investigation of patients with DD/ID obtained by using a combination of the MLPA sets for subtelomere aberrations and microdeletion syndromes followed by the confirmation of the aberrant results by the region-specific MLPA kits. The use of two subtelomeric kits per patient and investigation of all aberrations by follow-up sets has reduced the rate of false positive and negative results and improved the diagnostic yield. The relatively low cost, simplicity and reliability makes MLPA an effective first-tier cytogenetic diagnostic test for screening large cohorts of DD/ID patients.

## Background

Developmental delay (DD)/intellectual disability (ID) is a common problem, affecting 1-3% of the population. It has been estimated that one-half of the cases are due to genetic factors and chromosome aberrations have long been recognized as the most common cause of DD/ID [1]. High-resolution G-banded karyotyping and fluorescence *in situ* hybridization (FISH) studies using probes targeted to the subtelomeres and/or known microdeletion loci were considered the gold standard for detecting cytogenetic aberrations [2,3]. The development and availability of multiplex ligation-dependent probe amplification (MLPA) and array based comparative genomic hybridization (arrayCGH) techniques for the accurate assessment of copy number changes at multiple loci has provided a better approach for subtelomere and microdeletions testing in routine settings [4,5]. The next-generation sequencing techniques (NGS) which includes whole genome sequencing (WGS) or whole exome sequencing (WES), that may detect single-nucleotide changes through the whole genome are now increasingly applied in clinical diagnostics [6,7]. A major challenge in the application of these new techniques is the efficient analysis of a large amount of generated data. In addition, high costs of these genetic tests make them unavailable in modest clinical settings.

Since MLPA is relatively fast, easily interpreted and cost-effective, it is still a method of choice for screening large cohorts of DD/ID patients in developing countries [8]. We present a diagnostic workflow that uses a combination of MLPA kits for the effective high throughput routine diagnostics of unselected DD/ID patients.

## Results

**Figure 1:**
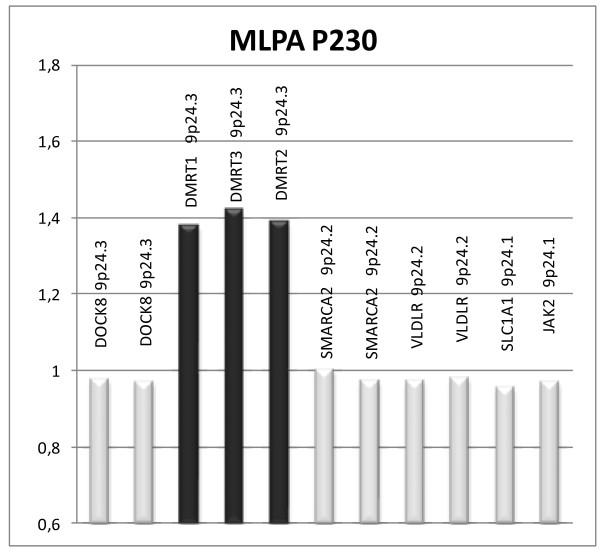
**MLPA P230 result of patient T4 with interstitial duplication 9p24.3.** The partial diagram shows abnormal peaks for *DMRT1, DMRT3* and *DMRT2* genes (peak range 1.37-1.42) and normal peaks for *DOCK8, SMARCA2, VLDLR, SLC1A1* and *JAK2* genes (peak range 0.95-1).

One hundred and fifty patients with unexplained DD/ID with or without dysmorphic features (DF) or congenital anomalies (CA) were included in the study. After exclusion of one false-positive result caused by maternal polymorphism, 21 (14%) of them were found to have chromosomal imbalances. There were 11 (7.3%) subtelomeric rearrangements. The results are summarized in Table 1. Subtelomere imbalances were detected in 9 patients by both probe sets, while in 3 patients (T2, T4 and T12) the imbalances were detected only by P036 set. In patient T2, MLPA SALSA P036 revealed a *de novo* 15q subtelomere deletion. Subsequent analysis with P070 showed normal result. The patient presented with severe short stature which is in agreement with the haploinsufficientcy of *IGF1R* gene located in the 15q subtelomere region. The ambiguous result was resolved by further analysis of healthy parents with P070. This revealed maternal polymorphism (dup 15qsubtel, duplicated signal for *TM2D3* gene) resulting in the false normal MLPA SALSA P070 signal in the patient. Follow-up MLPA with SALSA P291 additionally confirmed the presence of del 15qsubtel. In patient T4 MLPA SALSA P036 detected dup 9psubtel, but P070 analysis was again normal. MLPA follow-up set for 9p (P230) showed interstitial duplication that included only probe from SALSA P036 set (Figure 1). In patient T12 MLPA P036 showed del 4qsubtel. Subsequent MLPA SALSA P070 was normal. MLPA analysis with parental samples (P036, P070 and follow-up P264) revealed the deletion signals for *TRIML2* and *ZFP42* genes in the patient and in healthy mother, demonstrating that the imbalance is maternally transmitted polymorphism. In three patients (T5, T6, T7), the same abnormality was detected in the respective affected parent. In two cases with unbalanced translocations the origin of the unbalanced karyotype could be traced to the presence of a balanced translocation in a parent (T8; maternal and T9; paternal) detected by high-resolution G-banding. In the third case (T10) the subtelomeric translocation del12psubtel/dup22qsubtel was not detectable by high-resolution karyotyping. Using P245 kit we have additionally found 10 (6.6%) microdeletions (5 DiGeorge syndromes, two 17q21.31 microdeletions, one 15q24 microdeletion, one Prader-Willi/Angelman syndrome and one Langer-Giedion syndrome) (Table 2).

**Table 1:** Overview of the detected abnormalities found in patients with subtelomeric MLPA

**Patient**	**Age**	**Gender**	**DF**	**CA**	**P036**	**P070**	**Follow-up MLPA**	**Karyotype**	**Deletion size (Mb)**
T1	2y	F	+	+	del4p	del4p	P096	46,XX,del4p16.3 *	4,5
T2	2y1m	F	+	+	del15q	*x*2	P291	46,XX.mlpa 15q26.3(P291)x1	3 – 4
T3	3y	F	+	+	del22q	del22q	P188	46,XX.mlpa 22q13.3(P188)x1	1 – 4,6
T4	4y2m	M	+	-	dup9p	*x*2	P230	46,XY.mlpa 9p24.3(P230)x3	0,45 – 1,7 **
T5	3y11m	F	+	-	dupX/Yp	dupX/Yp	P018	46,XX.mlpa Xp22.32(P018)x3 mat	0,86 **
T6	1y4m	M	+	-	dupX/Yp	dupX/Yp	P018	46,XY.mlpa Xp22.32(P018)x3 mat	0,86 **
T7	2y4m	M	+	-	dupX/Yp	dupX/Yp	P018	46,XY.mlpa Yp11.32(P018)x3 pat	0,7
T8	11y9m	F	+	+	dup3p/del8q	dup3p/del8q	P208 P320	46,XX,der(18)t(3;18)(p26.1;q22.1)mat*	dup3p~6 del18q ~12,5
T9	2y4m	M	+	+	dup8p/del18q	dup8p/del18q	P208 P320	46,XY,der(18)t(8;18)(p23.1;q22.1)pat*	dup8p~10 del18q ~13,8
T10	7y11m	F	+	+	del12p/dup22q	del12p/dup22q	P230 P188	46,XX.mlpa 12p13.3(P230)x1,22q13.3(P188)x3	del12p 4,2–6,1 dup22q 4,6–6
T11	13y4m	F	+	+	del19p	del19p	P249	46,XX.mlpa 19p13.3(P249)x1,19p13.3(P249)x3	del ≤0,5 dup ≥2,7
*T12*	*5y10m*	*M*	*-*	*-*	*del4q*	*x2*	*P264*	*46,XY*	*-*

DF -dysmorphic features, CA -congenital anomalies, M -male, F -female.

* Additionally high resolution karyotyping.

** Intrachromosomal duplication.

**Table 2:** Overview of the detected abnormalities found in patients with microdeletion

**Patient**	**Age**	**Gender**	**DF**	**CA**	**P036**	**P070**	**P245**	**Follow-up MLPA**
M1	6m	F	+	+	*x*2	*x*2	del22q11	P250 del 14 probes at 22q11.23 ^a^
M2	1y1m	F	+	+	*x*2	*x*2	del22q11	P250 del 14 probes at 22q11.23 ^a^
M3	6y10m	M	+	+	*x*2	*x*2	del22q11	P250 del 14 probes at 22q11.23 ^a^
M4	2y10m	F	+	+	*x*2	*x*2	del22q11	P250 del 14 probes at 22q11.23 ^a^
M5	11m	M	+	+	*x*2	*x*2	del22q11	P250 del 14 probes at 22q11.23 ^a^
M6	15y6m	F	+	+	*x*2	*x*2	del17q21.31	P371 del 8 probes at 17q21.31
								P275 del 20 probes at 17q21.31 ^b^
M7	3m	M	+	+	*x*2	*x*2	del17q21.31	P371 del 8 probes at 17q21.31
								P275 del 20 probes at 17q21.31 ^b^
M8	5y8m	M	+	**-**	*x*2	*x*2	del15q24	P371 del 9 probes at 15q24.1 ^c^
M9	11y2m	M	+	**-**	*x*2	*x*2	del8q24.1	P371 del 10 probes at 8q24.1 ^d^
M10	17y2m	F	+	**-**	del15q11	del15q11	del15q11	P374 del 10 probes at 15q11.2 ^e^

DF -dysmorphic features, CA -congenital anomalies, M -male, F –female, LCR –low copy repeat.

^a^ Breakpoint between LCR-A i LCR-D, commonly deleted DiGeorge region.

^b^ Deletion of the CRHR1, IMP5 and MAPT genes.

^c^ Additionally high resolution karyotyping: 46,XY,del15q23q24.1.

^d^ Deletion of the TRPS1, EIF3H and EXT1 genes.

^e^ Deletion of the SNRPN and UBE3A genes.

**Figure 2:**
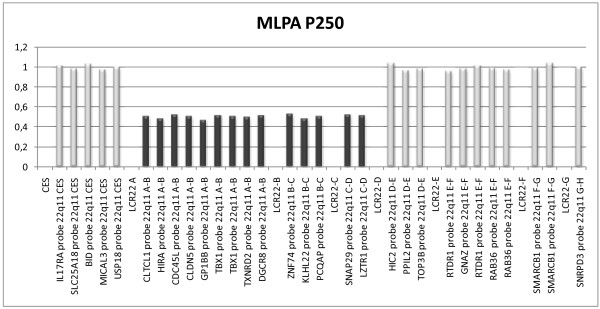
**MLPA 250 result of patient M1 with deletion 22q11.2.** The diagram shows typical deleted region between low-copy repeats (LCR) A and D.

All imbalances were verified by follow-up MLPA kits. In seven (63.6%) subtelomere cases and in all 5 cases of DiGeorge syndrome we were able to determine the approximate size of the imbalances (Figure 2, Table 1 and Table 2). In four (36.3%) subtelomere cases aberrations extended beyond the follow-up MLPA coverage. In three patients with subtelomere aberrations and in one patient with del 15q24 microdeletion we were able to visualise the imbalances by high-resolution karyotyping at 850-band level. Although aberrations of 3–5 Mb are presumably visible by high resolution chromosome banding, the case T10 (del12psubtel/dup22qsubtel) illustrates how some subtelomeric disorders are difficult to visualise by karyotyping due to their location in the distal G-negative staining region.

The costs of MLPA analysis using three or four kits in our laboratory (including reagents, import taxes and workload) are 600–800 EUR per patient. As the array CGH and NGS techniques are not available in clinical practice in our country, we would be obliged to perform these analyses abroad. The cost for DD/ID/Congenital anomaly arrayCGH in Europe is on average 1200 EUR and for NGS 3600 EUR (http://www.gendia.net). This is double or triple compared to our costs. Still, by using these techniques, there will be probably additional 6-8% improvement in detection rate of genomic rearrangements.

## Discussion

The improving resolution of molecular cytogenetic techniques increases the detection rate of cryptic chromosomal abnormalities in individuals with DD/ID [9]. Several studies have demonstrated the feasibility of arrayCGH or MLPA as first line diagnostic tests, replacing G-banding chromosome analysis in the detection of constitutional imbalances [8,10-12]. ArrayCGH and NGS techniques offer the highest diagnostic yield but are relatively expensive and laborious. Compared to them, MLPA is relatively fast and low cost and the application of follow-up MLPA kits represent a reasonable choice in designing a testing algorithm for DD/ID patients when new technologies are not available. Studies of unselected patients with DD/ID and normal karyotype using different strategy of MLPA subtelomere screening have identified pathogenetic imbalances in 2.9-5.3% of patients. These studies have used only one MLPA set [13-15], or a combination of two sets [16-18]. Abnormal results were subsequently tested by FISH, quantitative PCR or high resolution-CGH. There is no generally accepted diagnostic protocol, but the simultaneous use of two distinct kits of subtelomeric primer sets as a mean of independent confirmation is recommended by the manufacturer and now commonly accepted as a standard procedure. In our study subtelomeric imbalances were found in 11 (7.3%) patients. The incidence of subtelomere aberrations is higher than in previous studies, which illustrates the efficiency of using two ST-MLPA kits in all patients and evaluating further aberrant results with follow-up MLPA kits. Of 12 patients, three showed a single aberrant telomeric signal. These patients were further evaluated by investigating parental DNA and by using follow up kits. Two imbalances where shown to be true positive. Previous publications on the use of MLPA to detect subtelomere imbalances recommended that both different MLPA sets need to demonstrate concordance for an abnormal result in order to reduce the risk of detecting superfluous polymorphisms [16,17,19]. However, imbalances identified by a single probe may be true positives as exemplified by our patients. The false negative or positive result in one of the kits can be caused by the parental polymorphism or by the particular position of the imbalance that may stretch away from the second locus tested when the breakpoint lies between the two MLPA probes. Thus, single probe imbalances need further careful investigation.

The use of follow-up MLPA kits enables conformation and further delineation of chromosome imbalances. By using MLPA follow-up kits we were able to determine the approximate size in two thirds of subtelomeric imbalances. In addition, in one patient (T11) with distal subtelomere deletion at 19p, chromosome specific MLPA SALSA P249 revealed proximal duplication. This is the first study to use follow up kits for confirmation of the MLPA results and for establishing the approximate size of the imbalances. This approach was quite effective as we were able to confirm (T4) or detect (T11) two duplications that would probably be missed by FISH. One fourth of subtelomere imbalances were not true cryptic, as they were detectable by high resolution karyotyping at >800-band level. Our results confirm the observation by Jehe et al. that, although confirmatory tests using other technologies such as FISH or arrayCGH are welcomed, testing with MLPA kits in combination with high resolution karyotyping and/or revision of clinical findings is in most cases sufficient for establishing the diagnosis [8].

The incidence of common microdeletion/microduplication syndromes was 6.6%. In similar MLPA studies with SALSA P064 kit, detection rate varied from 5.8% to 14.1%, probably depending on the selection criteria of the patients [8,20]. All microdeletions were confirmed by the appropriate follow-up MLPA kits. In our study del 22q11.21 was the most frequent abnormality, found in 3.3% of patients and representing one fourth of all detected aberrations. This is in agreement with other studies where del 22q11.21 is also the most frequent aberration found in 1.5% to 7% of patients [8,20]. MLPA kit SALSA P250 additionally characterizes the size of del 22q11.21. All five patients had a typical proximal 3 Mb deleted region between low-copy repeats A and D. In one patient high resolution G-banding subsequently confirmed an intrachromosomal deletion at 15q23q24.1 indicating failure of standard karyotyping in studying some G-negative staining regions.

## Conclusions

We have presented the results of the investigation of patients with DD/ID obtained by using a combination of the MLPA sets for subtelomere aberrations and microdeletion syndromes, followed by the confirmation of the aberrant results by the region-specific MLPA kits. With the use of MLPA as the only molecular method and with the relatively simple strategy, we were able to detect clinically relevant aberrations in 14% of unselected patients with DD/ID.

The lower cost, simplicity and reliability of MLPA makes her an effective first-tier cytogenetic diagnostic test for screening large cohorts of DD/ID patients and a good alternative option for diagnostic cytogenetic laboratories where arrayCGH and new NGS technologies are not readily available.

## Material and methods

### Patients

This prospective study includes hundred and fifty patients referred because of DD/ID. All patients were evaluated by clinical geneticist and had a normal routine karyotype (450–500 band level resolution). Patients were not additionally clinically selected/subdivided on the basis the presence of DF, CA or severity of the DD/ID, thus enabling a broad screening regardless of the level of ID or the presence of associated abnormalities.

### Methods

We used two distinct MLPA kits, SALSA P036-E1 and SALSA P070-B1, for subtelomere screening and one MLPA kit for the 21 common microdeletion syndromes, SALSA P245-A2 (MRC Holland, Amsterdam, the Netherlands). Subtelomere analysis was performed by both kits in all patients. MLPA was carried out according to the manufacturer’s instructions. Amplification products were identified and quantified by capillary electrophoresis on an ABI 310 genetic analyzer, using GenMapper, vs 4.0 (Applied Biosistems, Foster City, CA, USA). The interpretation of fragment analysis, peak heights and areas of the amplified probes, were has been done using Microsoft Excel template. Data analysis was performed according to the manufacturer’s recommendation, using global or block normalization, respectively (MRC-Holland, Amsterdam, the Netherlands).

When an aberration was found, confirmatory testing was performed with follow-up MLPA kits with more probes in specific chromosomal region, which are able to confirm and specify the abnormalities in more detail. In addition, high-resolution G-banded karyotyping studies at a >800-band level were performed in all patients with newly discovered chromosomal imbalances.

## Abbreviations

DD/ID: Developmental delay/Intellectual disability; FISH: Fluorescence *In Situ* hybridization; MLPA: Multiplex ligation-dependent probe amplification; CGH: Comparative genomic hybridization; NGS: Next-generation sequencing; WGS: Whole genome sequencing; WES: Whole exome sequencing; DF: Dysmorphic features; CA: Congenital anomalies.

## Competing interests

The authors declare that they have no competing interests.

## Authors’ contributions

LMP developed the MLPA diagnostic analysis strategy, performed the cytogenetic and MLPA analysis and wrote the manuscript, IB initiated the study, coordinated the clinical analysis of the patients and revised the manuscript. KD and LO helped with data collection, processing and writing the manuscript. All authors have read and approved the final version of the manuscript.
